# Cute Communication: Can Cute Discourse Be Used in Risk Communication?

**DOI:** 10.3390/bs15101377

**Published:** 2025-10-10

**Authors:** Lu Zhang, Guohua Wang

**Affiliations:** College of Public Administration, Huazhong University of Science and Technology, Wuhan 430074, China; mpawin@hust.edu.cn

**Keywords:** Weibo, emergency government, cute discourse, risk communication effect

## Abstract

The development of social media has brought new challenges to government risk communication, and the public has put forward higher requirements for emotionality, novelty, and interactivity in communicating risk information. Based on this, the Chinese emergency government has adopted a new expression on social media platforms—cute discourse. The emergency government’s use of cute discourse to mitigate negative public emotions and enhance information attraction. Although there is a large number of studies on government risk communication on social media, there is a lack of research on the effectiveness of using this new expression. In this study, we analyzed the 11,152 emergency government’s posts on Weibo in China and assessed the impact of the use of cute discourse on risk communication effect under a mixed research method. The results show that within the range of sample values, the use and degree of the emergency government’s cute discourse improves communication effectiveness. Additionally, the driving effect of the use and degree of the emergency government’s cute discourse on risk communication varied across crisis types, risk stages, and risk themes. These results provide novel approaches and new perspectives for the study of governmental risk communication discourse.

## 1. Introduction

In modern society, risks are omnipresent, and risk communication in emergency management has become a crucial tool for addressing unforeseen events and maintaining social stability (Yudarwati et al., 2022). However, traditional risk communication methods, often characterized by seriousness and credibility, have limitations in appeasing public sentiment and attracting public attention (Choi, 2021; Y. Y. Guo et al., 2020). Meanwhile, government risk communication on social media is facing new challenges. The government not only needs to convey more accurate and complete risk information but also needs to consider the public’s demand for novelty, emotionality, and interactivity in this new era. Based on this, the emergency government has begun to use cute discourse on social media platforms (K. M. Li, 2021; Qiu, 2013), that is, integrating cute elements into discourse content and conveying information through gentle, soothing, and interesting expressions (Kronrod & Danziger, 2013).

Based on the existing literature (H. Y. Yang, 2023), this study defined the cute discourse of the government as an information expression in which the government, with “cuteness” as the core creative element, symbolically interprets public affairs and conveys political ideas through visual symbols, personified expressions, and emotional narratives. The core lies in reducing the defensiveness of public communication by leveraging the cute elements that are widely loved by the public, so that policy advocacy or national image construction can gain broader attention and emotional recognition. Among them, the word “cute” refers to an adjective with the meaning of “pretty” or “lovable”, and is used to describe people, animals, objects, or behaviors that can evoke feelings of affection, pleasure, and warmth (Kringelbach et al., 2016). Meanwhile, due to the multimedia functions of social media platforms, forms of cute discourse expression are diverse, including words, emojis, symbols, pictures, videos, and music.

This unique expression of emergency government discourse meets the needs of risk communication on social media platforms, which not only conveys information in a new way but also soothes negative public emotions (Neilson, 2018). This discourse shift has attracted the attention of some scholars. However, its specific impact on government risk communication has not yet been studied. We address the following in the subsequent sections: Does the use of cute discourse by the government have an impact on the effect of risk communication? If so, are there any differences in different risk scenarios? This study addresses these issues by examining whether the use of cute discourse by the Chinese emergency government on the Weibo platform affects the effectiveness of risk communication.

## 2. Literature Review

### 2.1. Government Risk Communication on Social Media

Over the past few decades, a large number of academic studies have discussed risk, disaster, or crisis communication (Haer et al., 2016; Z. Y. Li et al., 2021). In existing studies, risk communication is widely described as an interactive process of exchanging dangerous and unsafe information among individuals, groups, and institutions (Kim & Kreps, 2020). With the rapid development of technology, the places where risks and disasters are transmitted have shifted from offline to online. Social media, represented by Twitter, have become platforms for the government to convey risk information, and for the public to receive disaster information and express emotions to commemorate disaster victims. Increasingly, both central and local emergency governments have established official accounts on social media, using their platform to release authoritative risk information and respond to public concerns.

The objective of government risk communication emphasizes that, by releasing timely and accurate information on disaster events to the public, it can prompt the public to implement self-protection mechanisms, thereby reducing risks. Existing research emphasizes that various factors influence the effectiveness of government risk communication on social media, including personal (interpersonal relationships, culture, expectations, perception, identity, empathy ability, and emotions of anxiety) (Cummings et al., 2013) and government (information release methods, content, and time) factors (Twyman et al., 2008).

Despite its communication potential, the government’s use of social media faces notable challenges in the new era. The government not only needs to provide the public with more convenient and timely risk information but also needs to enhance the novelty, emotionality (Jacobson & Mascaro, 2016), and interactivity of the information content (Mergel, 2013). On the one hand, the threatening, unpleasant, and demanding characteristics of risk information (Slovic et al., 2004) can cause and amplify the public’s negative emotions (e.g., avoidance, frustration, anxiety, panic, and stress) (J. Li & Li, 2024; Martela et al., 2021), requiring the government to adopt positive discourse to soothe the public’s emotions. Specifically, these negative emotions can amplify the public’s assessment of the objective probability of risk occurrence, thereby weakening the benign transmission of risk information (Nan, 2017). On the other hand, the convenience of social media platforms has increased the frequency of the release of government risk information content. Repeated information dissemination has led to the phenomenon of public cognitive overload (Skurka et al., 2018), and the government needs to adopt more novel content to attract public attention. Repeated transmission of disaster information can lead the public to receive excessive information, causing mental fatigue (Dhir et al., 2018), and bring ineffective results to communication effectiveness. Therefore, disaster researchers stress that the government not only needs to disseminate complete risk information but also needs to use creative and expressive content and methods to attract public attention on social media.

Effective information content and release methods promote the effect of government risk information dissemination (Haer et al., 2016). In order to promote benign risk communication, the government has adopted various innovative content and methods on social media platforms to alleviate the public’s negative emotions (Xie et al., 2011) and burnout, which has also been the focus of scholars’ research in recent years. Existing research has found that, on the one hand, the government promotes persuasive information by providing corresponding emotional support (e.g., encouragement and sympathy) (Díaz et al., 2020) to the public (Ward & Budarick, 2021). Specific measures include the use of humorous discourse (Hämpke et al., 2022) and cute discourse (Cheng & Lee, 2019). On the other hand, the government takes advantage of the multimedia functions provided by social media platforms to enhance the interest of risk information and its persuasive effect through visual effects such as pictures/illustrations, data visualization, and photos.

### 2.2. Cuteness in Public Messaging

Cuteness, defined as evoking feelings of happiness and warmth in an attractive way, is an informal language style (Hecht & Horowitz, 2015). Existing research mainly falls into two categories, one of which usually links to concepts such as innocence and naivety (Borgi et al., 2014). This type of cuteness stimulates the observer’s neural circuits (Kringelbach et al., 2008) by means of physical features (e.g., round face, big head, and big eyes), triggering attention, intimacy (Borgi et al., 2014), caring (Hashizume & Kurosu, 2019), preference (Esposito et al., 2014), trust (K. Yang, 2023), and protective behavior (Zhang et al., 2022). Whimsical cuteness, another type of cuteness, is often associated with functional traits (e.g., unconventional, funny, playful, and humorous) (Nenkov & Scott, 2014). This type of cuteness has a spillover effect and can be used to facilitate information dissemination (Sherman & Haidt, 2011), public interaction (Kuraguchi & Ashida, 2015) and emotional expression (Scott & Nenkov, 2016) through interesting and diverse designs (Qin & Wang, 2024). For example, sound symbols, pictures (Lieber-Milo et al., 2024), and even emoticons promote relationships and connections on social media platforms (Lee, 2024). Based on the existing literature, this study defined the cute discourse of the government as an information expression in which the government, with “cuteness” as the core creative element, symbolically interprets public affairs and conveys political ideas through visual symbols, personified expressions, and emotional narratives. Its forms of discourse expression are diverse, including words, emojis, symbols, pictures, videos, and music.

### 2.3. Cuteness and Risk Communication on Social Media

In the context of social media, cute elements are also considered, by the public and government, to have become a common way of information dissemination and social sharing. In risk communication, the audience is often resistant to negative information, while cute discourse can convey feelings of warmth, intimacy, sympathy, and relaxation (Fiske, 2020). These feelings not only reducing the psychological resistance of the audience (Cui, 2024) but also by stimulating positive emotions (Lv et al., 2021). Positive emotions can trigger the brain’s response to happiness and generate emotional resonance (Batson et al., 2005). This emotional resonance not only enhances rational communication by soothing the public’s anxiety and panic caused by risks (Zhang et al., 2022) but also makes people more willing to accept and share such content (Aragón & Bargh, 2018). It can act as an icebreaker in digital interaction, shorten the distance between the government and the public, and promote the two-way communication of risks (Wong et al., 2021).

In addition, cuteness attracts people’s favor and likes (Peng et al., 2022; Yim et al., 2024), making them actively pay attention to and browse risk information. The repeated risk information warnings and dissemination on social media platforms consume the public’s patience and are prone to causing information fatigue. The entertaining content and diverse forms of cute discourse meet the interest demands of the public, especially younger groups, alleviate the phenomenon of information burnout, and increase information-sharing behavior (Hedman, 2016). The multimedia functions of social media platforms have enriched the communication methods of emergency governments. By integrating multiple modalities, such as language, images, emojis, and videos, risk information can be presented in an abundant and intuitive way. This communication approach can also enhance the audience’s sense of participation (Ngai, 2023), making them more actively involved in the process of risk communication. Accordingly, the following hypotheses are proposed:

**Hypothesis** **1.**  *On social media, the use of the emergency government* *’s cute discourse is positively correlated with the risk communication effect.*

Meanwhile, existing research has found that a high degree of cute discourse can amplify the effects of utilizing cuteness but may clash with certain themes (Steinnes et al., 2019). Specifically, the improper use of cuteness, to a certain degree, by the emergency government in some risk scenarios, can have negative effects (Shi et al., 2021). For instance, when emergency governments incorporate cute elements in leisure and entertainment content, it can increase the audience’s willingness to share, and a higher degree of cuteness can expand this willingness (Septianto & Kwon, 2022). However, when the cuteness discourse is applied to severe disasters, it may accelerate the public’s aversion (Shi et al., 2021). Therefore, the government needs to reasonably distinguish risk scenarios and adjust the use and degree of discourse to exert the positive impact of cute discourse and reduce the negative impact of cute discourse. Accordingly, the following hypotheses are proposed:

**Hypothesis** **2.**  *On social media, the degree of the emergency government* *’s cute discourse is positively correlated with the risk communication effect.*

**Hypothesis** **3.**  *The effect of the emergency government* *’s use of cute discourse on the risk communication effect varies across different crisis types, risk stages, and risk themes.*

**Hypothesis** **4.**  *The effect of the degree of the emergency government* *’s cute discourse on the risk communication effect varies across different crisis types, risk stages, and risk themes.*

Despite the growing body of work on cute elements and risk communication, shortcomings remain. On the one hand, while many studies acknowledge the influence of cute elements on social media information dissemination, few provide empirical evidence on their effects in risk communication. On the other hand, although scholars generally agree that the effect of using cute discourse is affected by different risk scenario factors, empirical testing is scarce. In view of this, this study not only examines whether the use of the government’s cute discourse affects the effectiveness of risk communication, but also examines its driving effect on the risk communication effect under different risk scenarios.

This research has three key contributions. First, previous studies have focused on the effect of using cute discourse in conventional scenarios, while this study reveals the impact of cute discourse on the communication effect in risk scenarios, providing a valuable research perspective for the use of new discourse in risk scenarios. Second, the effect of the cute discourse centered on the emergency government on social media platforms was explored, providing a basis of data for the research on government discourse in cyberspace. Third, the research results provide practical insights into the use of governmental cute discourse under different risk scenarios and offer supportive data for the use of governmental cute discourse in practice.

## 3. Materials and Methods

### 3.1. Data Sources

Among the existing social media platforms, Weibo in China was chosen for this study to investigate the impact of the use and degree of the emergency government’s cute discourse. The selection of the platform and samples was mainly based on the following considerations. First, Weibo was chosen as an important representative of social media platforms that Internet users use to browse risk events and make interactive comments; second, based on the “Government Weibo Influence Report”^1^, 15 emergency government accounts were selected according to the highest order of dissemination and influence (Table 1). Finally, in accordance with international news-gathering regulations, the period from 10 July 2023 to 13 August 2023 was selected as the data’s time range to observe users’ risk communication (Lovejoy & Saxton, 2012); by the end of the study, 11,152 posts were collected through Python (version 3.10) software.

### 3.2. Methods

This study used a mixed research method that combined a coding procedure and a regression model to analyze the impact of the use and degree of the emergency government’s cute discourse. The steps involved are discussed below.

#### 3.2.1. Coding Procedure

In this study, content analysis was chosen as the coding method, and two Chinese research assistants were trained to code the content sentence-by-sentence. After the training, they were asked to code 1000 of the 11,152 posts published by the emergency government and calculate the consistency reliability. Until a high level of agreement reliability was achieved, the research assistants determined the final coding rules and coded the content of the 11,152 samples. After extracting the concepts and categories, the research assistants compiled two main categories. The two main categories divided the independent variables into two parts: the use of the emergency government’s cute discourse and the degree of the emergency government’s cute discourse. The reason for the division into two independent variables was that both are important factors influencing public interaction, but they play different roles in different scenarios. The coded Cohen’s κ coefficients for the two independent variables were 0.986 and 0.985, respectively, indicating a high and stable correlation.

#### 3.2.2. The Least Squares Regression Model

This study made use of the ordinary least squares regression model in Stata (version 15.1) software to test the likelihood of positive results for two independent variables. In Equations (1) and (2), Y represents the risk communication effect. β_0_ and β_3_ represent the intercept vector, while β_1_, β_2_, β_4_, and β_5_ represent the estimated parameters of each variable. X_1_ represents the emergency government’s use of the cute discourse, and X_2_ represents the degree of the emergency government’s cute discourse. X_i_ represents the other explanatory variables, and ε_0_ and ε_1_ are random disturbances:

(1) $$Y=\beta_{0}+\varepsilon_{0}+\beta_{1}X_{1}+\beta_{2}X_{i}$$

(2) $$Y=\beta_{3}+\varepsilon_{1}+\beta_{4}X_{2}+\beta_{5}X_{i}$$

### 3.3. Variables

#### 3.3.1. Independent Variables

In this study, the use and the degree of the emergency government’s cute discourse were taken as independent variables. Existing research indicates that the presentation forms of government cute discourse on Weibo include words, emojis, symbols, pictures, videos, and music (J. Su, 2025). Therefore, based on the existing literature, this study measured cute discourse according to the coding criteria of cute vs. non-cute (Steinnes et al., 2019; Q. Q. Su & Li, 2023). When examining whether the emergency government’s Weibo posts use cute discourse, if the content of the blog contained cute elements, it was considered to have used cute discourse and recorded as 1; otherwise, it was recorded as 0 (Table 2).

Existing studies primarily employ the ratio of vocabulary size to text length to assess the degree of vocabulary richness (Fergadiotis et al., 2019; Wang & Liu, 2018). Additionally, scholars have begun using the ratio of shape area to colored area as another important attribute to measure the cuteness of graphics (Laohakangvalvit & Ohkura, 2018). Therefore, according to the existing research findings (Fergadiotis & Wright, 2016) and characteristics of Weibo, this study measured the degree of the government’s cute discourse using the proportion of cute content in the total content. The calculation of the degree of government cute discourse is presented in Table 3.

#### 3.3.2. Dependent Variables

Based on the existing literature, this study chose the risk communication effect as the dependent variable (Boholm, 2019). Risk communication effect is an important index system composed of information transmission (Jiang et al., 2021; Zhu et al., 2022), information dissemination (MacKay et al., 2021), subject interaction, governance risk, and other factors. Scientifically and accurately measuring the effect of risk communication is an important way to improve the efficiency of government discourse communication. The entropy method, as an objective assignment method, measures weights based on the uncertainty of random variables in information theory (Bai et al., 2022), and it can solve index weights without interference from human factors. It originated from the concept of information entropy and is currently widely applied in economics and environmental sciences (Xu et al., 2021). Therefore, based on existing studies and risk communication indicators (Bai et al., 2024), this study used the value entropy method to measure risk communication effect indicators. The selected indicators include risk information transmission, risk information dissemination, risk interaction, and risk governance effect. The specific variables and weights are shown in Table 4 below.

#### 3.3.3. Control Variables

Risk disaster researchers believe that the effectiveness of risk communication is also affected by other factors, such as the information source and account fans. Therefore, this paper introduces other factors that affect the effect of risk communication as control variables, which mainly include six variables: information source, account fans, intergovernmental interaction, account concerns, account friends, and account years. At the same time, blog characters are introduced as control variables in the robustness test.

Based on the principles of data integrity and availability, logarithmic processing was carried out on the dependent variables (risk communication effect) and control variables (account fans, account concerns, account friends, and account years). Stata (version 15.1) software was used to perform descriptive statistics on each variable, as shown in Table 5.

## 4. Results

Before regression analysis, it was necessary to perform a test to avoid multi-collinearity. A variance inflation factor (VIF) was used in this test. The results showed that the VIF value of each variable was less than ten, and the average value of the VIF was less than five, indicating that there was no serious multi-collinearity problem between the variables.

### 4.1. Baseline Regression Analysis

After conducting the test, the data were regressed (Table 6). The results were as follows: The emergency government’s use of cute discourse had a positive impact on the risk communication effect (β > 0, *p* < 0.05). Additionally, within the range of sample values, the degree of the emergency government’s cute discourse had a positive impact on the risk communication effect (β > 0, *p* < 0.001). Thus, hypotheses 1 and 2 were verified. This means that, on social media, the emergency government’s use of cute discourse, especially when used to a high degree, promotes risk communication effectiveness. These conclusions are due to a variety of factors, including the appeal of this new discourse to Internet users and the soothing effect of the cute element on emotions. For instance, cute discourse can serve as a break from the intensity of emergency information, providing a moment of levity that allows internet users to better absorb and process the information. Moreover, the utilization of cute discourse may foster a sense of trust and acceptance towards risk information, thereby enhancing the overall effectiveness of communication. However, it is important to recognize that the emergency government has moderately used cute discourse on social media platforms. The average degree of the emergency government’s cute discourse in the selected sample is 0.0885 (mostly low-level cute discourse). Therefore, we found that within the value range of different degrees of the government’s cute discourse (0–0.7273), the degree of the government’s cute discourse can promote the risk communication effect.

### 4.2. Counterfactual Test

To ensure the rationality of the regression model and accuracy of the estimated results, this study tested the robustness of the results by adding new control variables, Winsorizing dependent variables (Theriault et al., 2024), and replacing the dependent variables. The number of blog characters was added as a new control variable to the regression model for testing. The regression results were significant, indicating that the conclusions remained robust. In contrast, this study tested the new dependent variables by treating the extreme values of the dependent variables at the 1% and 99% quantiles (Zou & Xiong, 2023). Numbers less than 1% were assigned a value of 1%, and numbers greater than 99% were assigned a value of 99%. Then, this study recalculated the regression results of the new dependent variables. The regression results were significant, indicating that the use and degree of the emergency government’s cute discourse still had a positive effect on the risk communication effect. Finally, we replaced the effect of risk communication with the number of reads (Jiang et al., 2021) and estimated the influence of the use of cute discourse on the number of reads as a robustness test. The results are significant, indicating that the conclusion remains robust (Table 7).

## 5. Heterogeneity Analysis

### 5.1. Crisis Type Heterogeneity Test

In the above analysis, all samples are treated as homogeneous rather than heterogeneous. As an important factor affecting the emergency government’s risk discourse, it is necessary to consider the impact of crisis types. According to the existing literature, this study coded the crisis type and divided it into five categories (non-crisis, natural disaster, social accident, public health, and social security event) to investigate the differences in the use and degree of the emergency government’s cute discourse (Table 8).

The results show that (1) the use of the emergency government’s cute discourse has different effects on risk communication effect under different crisis types. The use of the emergency government’s cute discourse had a positive impact on the risk communication effect in non-crisis and natural disaster events, but had no significant impact on social accident, public health, and social security events. This difference is due to the expressive purpose of the post content. In non-crisis events, the use of the emergency government’s cute discourse can satisfy its need for information delivery and cordiality enhancement. In natural disaster events, the impact of use of the emergency government’s cute discourse is complex. The government can soothe public sentiment through the warmth attributes of cute discourse. However, excessive entertainment elements may lead to public aversion. Therefore, although the government can use cute discourse, it needs to consider the degree of cute discourse use in natural disaster events. The use of cute discourse in this case is elaborated in the following part of this study. Furthermore, since social accident, public health, and social security events have the characteristics of human factors and a large scope of harm, improper use of communication discourse in these incidents can generate a greater negative impact, so the government tends to use traditional discourse to convey a more effective message in these cases. Therefore, cute discourses are not suitable for use in these events.

(2) The degree of the emergency government’s cute discourse has different effects on the risk communication effect under different crisis types. The degree of cute discourse had a positive impact on the risk communication effect in non-crisis events, had a negative impact in natural disaster events, and had no significant impact on social accident, public health, and social security events. This difference is due to a combination of the degree of cute discourse and the purpose of the information. High levels of cute discourse are more entertaining, attractive, and effective when used in non-crisis events than in crisis events. Therefore, high levels of cute discourse are not suitable for use in natural disasters, social accident, public health, and social security events.

(3) What is surprising is that, after the inverted U-shaped curve test, the degree of cute discourse had a nonlinear influence on the risk communication effect in natural disaster events. When this study observed the effect of the use and degree of government cute discourse in natural disaster events, it was found that the coefficient results had opposite signs. The use of cute discourse had a positive impact, but the degree of cute discourse had a negative impact on the risk communication effect. In natural disaster events, the effect of the degree of governmental cute discourse on the risk communication effect showed a result of increasing first and then decreasing, and the impact of the risk communication effect is best at the degree of 0.08.

Based on the research content of the inverted U-shaped curve (Ahmadova et al., 2022), the occurrence of this phenomenon is due to the nonlinear influence brought about by the increase in cuteness. There is controversy in the existing literature regarding the effect of using cute discourse in crisis scenarios. On the one hand, scholars generally believe that the use of cute discourse has a positive impact on stimulating positive emotions and interests of recipients and promoting communication (Xiang & Park, 2023). On the other hand, many scholars also think that the use of cute discourse has a negative impact by weakening the public’s perception of severity (Xiang & Park, 2023) and causing public aversion (Shi et al., 2021). When the two opposing viewpoints are put together, they may both be correct. That is to say, the use of cute discourse may have positive and negative impacts on the risk communication effect, depending on the degree of cuteness. When the degree of cuteness reaches a critical point, the high degree of cuteness eventually turns into a negative result. Therefore, in natural disasters, the government’s use of endearing words with a degree value lower than 0.08 is more conducive to communication effectiveness.

### 5.2. Risk Stage Heterogeneity Test

The rapid development of internet technology requires the government to choose appropriate discourse modes, so as to more accurately meet the needs of the public for information at different risk stages. Accordingly, we divided the risk stages of the blog content into four categories (pre-risk stage, initial stage, maintenance stage, and assessment stage) (Y. N. Guo et al., 2025) to investigate the influence of the use and degree of the emergency government’s cute discourse on communication effect in different risk stages.

As shown in Table 9, the use and degree of the emergency government’s cute discourse have different effects on the risk communication effect under different risk stages. The use and degree of the emergency government’s cute discourse had a positive impact on the communication effect in the pre-risk and maintenance stages, had a negative impact in the initial stage, and had no significant effect in the assessment stage.

This difference is due to different stages of risk. In the pre-risk and maintenance stage, the use of cute discourse meets the communication needs of the emergency government. In the pre-risk stage, the use of the emergency government’s cute discourse can change the single communication mode to attract the online public, so as to meet the government’s purpose of better and faster information dissemination. In the maintenance stage, risk events developed to the middle or late stage and were initially controlled; so, at this time, the government not only needs to maintain close contact with the public to correct inappropriate or wrong information but also needs to convey mild and positive discourse to guide the public’s positive emotions. The use of cute discourse can help soothe the public’s negative emotions and promote rational thinking, so it has a positive effect at this stage. However, in the initial stage of risk occurrence, the use of cute discourse will affect the public’s sad emotions and cause resistance to the communication effect. In the initial and assessment stages of risk occurrence, governments tend to provide simple, reliable, searchable, consistent, and timely information in response to media and public concerns. The use of cute discourse conflicts with the professionalism and authority of the information delivered, so it is not suitable for the initial stage and assessment stage.

In different risk stages, the influence of the degree of cute discourse is more obvious, mainly because the high degree of cute discourse intensifies the impact on risk communication. The warm attribute of cute discourse has a more significant positive impact in the pre-risk and maintenance stages, but a high degree of cute discourse will deepen the damage to information authority and feasibility in the initial and assessment stages, so it will lead to more serious negative effects.

### 5.3. Risk Theme Heterogeneity Test

Due to the difference in risk content, the government also has different ways of delivering different risk content (L. F. Li et al., 2021). Therefore, it is necessary to analyze the differences in the communication effectiveness of different risk themes (L. F. Li et al., 2020). Based on the existing literature (Chen et al., 2020), this study codes the risk content, which is divided into five categories (disaster rescue, risk policy, risk science popularization, spirit publicity, and interactive leisure information), to investigate the differences in the use of cute discourse under different risk themes.

As shown in Table 10, the use and degree of the emergency government’s cute discourse have different effects on the risk communication effect under different risk themes. The use and degree of cute discourse have a positive impact on the communication effect in risk popularization and interactive leisure information, have a negative impact in disaster rescue information, and have no significant effect in risk policy and spirit publicity information. This difference is due to the fact that the theme of information influences the government’s choice of discourse mode. The government is more inclined to adopt serious and accurate traditional discourse in disaster rescue, spiritual publicity, and risk policy information, and is more inclined to adopt amiable and attractive discourse in risk popularization and interactive leisure information (You et al., 2015). Therefore, cute elements are more suitable for promoting emotional reactions and public responses in risk popularization and interactive leisure information, and they are not applicable to disaster rescue, spirit publicity, and risk policy information.

High levels of cuteness aggravated these differences, mainly due to the information subject’s choice of cuteness. The high degree of discourse in risk popularization and interactive leisure information can increase the discourse’s entertainment and emotion, thus increasing public interaction. The high degree of cute discourse will accelerate the collapse of authoritative information, so it is not suitable in areas of disaster rescue, spirit publicity, and risk policy information.

## 6. Conclusions

This study uses two mixed methods to investigate the actual impact of the use and degree of the emergency government’s cute discourse on the risk communication effect, and draws the following conclusions: On the one hand, within the range of sample values, the use and degree of the emergency government’s cute discourse can promote a good risk communication effect, and this conclusion is robust. On the other hand, the impact of the use and degree of cute discourse on the risk communication effect is significantly different under different crisis types, risk stages, and risk themes.

In order to improve the communication effect, this paper puts forward three suggestions: First, the emergency government should further promote the construction of new risk discourses, appropriately use cute discourse, and use a high degree of cute discourse to enhance the affinity and interest of risk communication. Second, the government should use cute elements to guide the public to generate positive emotion by arousing emotional resonance and increasing the risk discourse attractiveness to increase the risk information transmission range. Third, in the process of using cute discourse, the government should take differentiation measures under different risk scenarios. Facing different crisis types, the government should use cute discourse in non-crisis and natural disaster events. Increasing the degree of cute discourse can help deepen its beneficial effect on risk communication in non-crisis events. Facing different risk stages, the government should use cute discourse in the pre-risk and maintenance stages of risk occurrence. Increasing the degree of cute discourse can help deepen its beneficial effect on risk communication in the above two stages. Facing different risk themes, the government should use cute discourse in risk science popularization and interactive leisure information. Increasing the degree of cute discourse can help deepen its beneficial effect on risk communication in the above two areas.

The conclusion can be used as a reference for the research into new risk discourse. However, there are two shortcomings in this study. On the one hand, due to the limitation of the research content, this study lacks research on the combined influence of more than two factors (e.g., under different crisis types and different risk stages). On the other hand, since this study uses Weibo, a social media platform in China, as an example, its conclusion is special (cute words have higher acceptability on social media) so whether this conclusion can be applied to other channels of risk communication (e.g., official websites) needs further study. The above problems are the focus of future research.

## Figures and Tables

**Table 1:** Basic information about the emergency management government.

Number	Name	Department	Collected Posts
1	China fire and rescue	Ministry of emergency management fire and rescue	1000
2	Ministry of emergency management	Ministry of emergency management	763
3	HuNan fire and rescue	Hunan provincial fire rescue corps	978
4	HeNan fire and rescue	Henan provincial fire rescue corps	1011
5	ShanDong fire and rescue	Shandong provincial fire rescue corps	557
6	TianJin fire and rescue	Tianjin provincial fire rescue corps	961
7	GuangXi fire and rescue	Guangxi Zhuang Autonomous Region fire rescue corps	609
8	JiangXi fire and rescue	Jiangxi provincial fire rescue corps	799
9	JiangSu fire and rescue	Jiangsu provincial fire rescue corps	1017
10	YunNan fire and rescue	Yunnan provincial fire rescue corps	749
11	AnHui fire and rescue	Anhui provincial fire rescue corps	700
12	XinJiang fire and rescue	Xinjiang seismological bureau	753
13	LuoYang fire and rescue	Luoyang City fire rescue detachment	428
14	SanMing fire and rescue	Sanming City fire rescue detachment	547
15	China earthquake networks rapid report	China earthquake networks center	280

**Table 2:** Coding framework and examples of the government’s use of cute discourse.

Level 1 Indicator	Secondary Index	Example	Encoding
The government did not use cute discourse	Discourse that does not contain cute elements	# See JiangSu fire and rescue # Have a happy weekend. Good night	0
The government used cute discourse	Discourse that contains cute elements (Cute words; cute emojis; cute symbols; cute pictures; cute videos; and cute music)	Onomatopoeia and nickname; cartoon emojis and cute animal emojis; (^^); cute animal pictures and cartoons; animated and cute animal videos; and cute lyrics and children’s songs	1

**Table 3:** Calculation of the degree of cute discourse.

Blog Content Form	Exemplify	Numerical Value
Text + Picture	The degree of cute discourse = 1/2(Number of cute words/Total word count) + 1/2 (Cute content/Total content of the picture)	[0,1]
Text + Video	The degree of cute discourse = 1/2(Number of cute words/Total word count) + 1/2 (Video cute content/Total content of the video)	[0,1]
Text + Link	The degree of cute discourse = 1/2(Number of cute words/total word count) + 1/2 (Link cute content/Total content of the link)	[0,1]

**Table 4:** Indicator system of risk communication effect.

Level 1 Indicators and Weight	Secondary Index and Weight	Measurement	Indicator Properties
Information transmission effect (0.1071)	Account posts (0.0133)	The number of content posted by the account	+
	Information integrity (0.0101)	The proportion of event content elements (who, when, where, why, what, and how)	+
	Information timeliness (0.0003)	The time-lag between the occurrence of an event and the publication of the blog post	−
	Information pertinence (0.0373)	Content containing risk information is marked as 1, otherwise marked as 0	+
	Information usefulness (0.0461)	The number of comments that contain words such as acceptance	+
Information dissemination effect (0.4685)	Public acceptance (0.0710)	The number of comments that contain words such as understanding	+
	Public trust (0.0579)	The number of comments that contain words such as trust	+
	Public favorability (0.1039)	The number of comments expressing positive images of the government	+
	Public recognition (0.0324)	The number of comments expressing approval	+
	Emotional exposure (0.0002)	The number of positive public comments minus negative comments	+
	Emotional guidance (0.0107)	The number of likes	+
	Public attention (0.0365)	The number of comments that contain words such as attention	+
	Recommendation behavior (0.0487)	The number of comments that contain @	+
	Search behavior (0.0792)	The number of comments	+
	Supportive behavior (0.0280)	The number of shares	+
Interaction effect (0.3393)	Internal coordination (0.1022)	The number of comments from other governments	+
	Internal cooperation (0.0467)	The number of shares from other governments	+
	Professional information reference (0.0140)	The number of comments that contain words such as professional	+
	Public interaction (0.1764)	The number of replies from accounts	+
Risk governance effect (0.0851)	Emotional pacification (0.0499)	(Negative comments/total comments) < 10% marked as 1; otherwise marked as 0	+
	Rumor processing (0.0220)	The number of comments that contain words such as explaining the rumor	+
	Experience learning (0.0132)	The number of comments that contain words such as experience	+

Compiled by the author. + indicates a positive indicator.

**Table 5:** Descriptive statistics of the main variables.

Variable Name	Definition	Obs	Mean	Std. Dev.	Min	Max
Use of cute discourse by government (USE)	0 = No; 1 = Yes	11,152	0.7489	0.4337	0	1
Degree of cute discourse by government (DEGREE)	The degree of cute discourse	11,152	0.0885	0.1198	0	0.7273
Risk communication effect	The effectiveness of risk communication	11,152	0.1008	0.0344	0.0120	0.4753
Information source	1 = National government; 2 = Provincial government; 3 = City level government	11,152	1.9904	0.5973	1	3
Account fans	The number of account fans	11,152	5.0627	0.8503	3.2308	7.0881
Intergovernmental interaction	Whether to use @ in the post: 0 = No; 1 = Yes	11,152	0.1604	0.3670	0	1
Account concerns	The number of accounts that this account actively follows.	11,152	6.6859	0.5331	5.6021	7.4872
Account friends	The number of account friends	11,152	6.3351	0.5172	5.4116	7.1952
Account year	The number of years of account operation	11,152	2.3104	0.3013	1.3863	2.4849
Blog characters	The number of characters per blog post	11,152	4.5169	0.6465	1.3863	8.4286
Crisis type	0 = non-crisis event; 1 = Natural disaster event; 2 = social accident event; 3 = public health event; 4 = social security event	11,152	1.1962	0.8332	0	4
Risk stage	1 = pre-risk stage; 2 = initial stage; 3 = maintenance stage; 4 = assessment stage	11,152	1.9179	0.8662	1	4
Risk theme	1 = disaster rescue; 2 = risk policy; 3 = risk science popularization; 4 = spirit publicity; 5 = leisure interactive	11,152	2.5934	1.4496	1	5

**Table 6:** Benchmark regression results.

Variables	Risk Communication Effect *	
USE	0.02 * (0.01)	
DEGREE		0.16 *** (0.03)
Information source	0.06 *** (0.01)	0.06 *** (0.01)
Account fans	0.01 (0.01)	0.01 (0.01)
Intergovernmental interaction	−0.05 *** (0.01)	−0.05 *** (0.01)
Account concerns	0.03 (0.02)	0.03 (0.02)
Account friends	0.01 (0.02)	0.01 (0.02)
Account year	−0.06 *** (0.01)	−0.06 *** (0.01)
Constant	−2.67 *** (0.09)	−2.70 *** (0.09)
Controls	Yes	Yes
N	11,152	11,152

* The empirical results are calculated by Stata (version 15.1) software. * *p* < 0.05, and *** *p* < 0.001. Standard errors are in parentheses.

**Table 7:** Counterfactual test.

Variables	(1) Add New Variable		(2) Winsorize Treatment		(3) Replace the Dependent Variables	
USE	0.02 ** (0.01)		0.01 ** (0.01)		0.06 *** (0.02)	
DEGREE		0.15 *** (0.03)		0.14 *** (0.02)		1.35 *** (0.08)
Controls	Yes	Yes	Yes	Yes	Yes	Yes
N	11,152	11,152	11,152	11,152	11,152	11,152

The empirical results are calculated by Stata (version 15.1) software. ** *p* < 0.01, and *** *p* < 0.001. Standard errors are in parentheses.

**Table 8:** Heterogeneity analysis (1): under different crisis types.

Variable	Non-Crisis Event		Natural Disasters		Social Accident		Public Health		Social Security	
USE	0.0845 *** (0.0255)		0.0363 *** (0.0095)		0.0005 (0.0101)		0.0608 (0.0948)		0.0560 (0.0671)	
DEGREE		0.9429 *** (0.0744)		−0.1654 *** (0.0363)		0.0255 (0.0352)		−0.1831 (0.3238)		0.0018 (0.2272)
Controls	Yes	Yes	Yes	Yes	Yes	Yes	Yes	Yes	Yes	Yes
N	2572	2572	4136	4136	4231	4231	110	110	103	103

The empirical results are calculated by Stata (version 15.1) software. *** *p* < 0.001. Standard errors are in parentheses.

**Table 9:** Heterogeneity analysis (2) under different risk stages.

Variables	Pre-Risk Stage		Initial Stage		Maintenance Stage		Assessment Stage	
USE	0.08 *** (0.02)		−0.03 *** (0.01)		0.07 *** (0.02)		−0.04 (0.04)	
DEGREE		0.53 *** (0.05)		−0.23 * (0.09)		0.26 *** (0.05)		−0.08 (0.18)
Controls	Yes	Yes	Yes	Yes	Yes	Yes	Yes	Yes
N	4163	4163	4258	4258	2215	2215	516	516

The empirical results are calculated by Stata (version 15.1) software. * *p* < 0.05, *** *p* < 0.001. Standard errors are in parentheses.

**Table 10:** Heterogeneity analysis (3) under different risk themes.

Variable	Disaster Rescue		Risk Policy		Risk Science Popularization		Spirit Publicity		Interactive Leisure	
USE	−0.04 *** (0.01)		−0.01 (0.05)		0.03 * (0.01)		−0.08 (0.04)		0.08 *** (0.03)	
DEGREE		−0.27 *** (0.08)		0.07 (0.17)		0.21 *** (0.04)		−0.44 (0.25)		1.01 *** (0.09)
Controls	Yes	Yes	Yes	Yes	Yes	Yes	Yes	Yes	Yes	Yes
N	4170	4170	468	468	4166	4166	422	422	1926	1926

The empirical results are calculated by Stata (version 15.1) software. * *p* < 0.05, *** *p* < 0.001. Standard errors are in parentheses.

## Data Availability

Data source, China fire and rescue: https://weibo.com/u/3549916270 (accessed on 6 September 2023); Ministry of emergency management: https://weibo.com/u/5342220662 (accessed on 14 March 2023); HuNan fire and rescue: https://weibo.com/u/2648904693 (accessed on 6 September 2023); HeNan fire and rescue: https://weibo.com/u/2106558073 (accessed on 6 September 2023); ShanDong fire and rescue: https://weibo.com/u/3605364617 (accessed on 6 September 2023); TianJin fire and rescue: https://weibo.com/u/2085621483 (accessed on 6 September 2023); GuangXi fire and rescue: https://weibo.com/u/2057392851 (accessed on 6 September 2023); JiangXi fire and rescue: https://weibo.com/u/1986679210 (accessed on 6 September 2023); JiangSu fire and rescue: https://weibo.com/u/2258717480 (accessed on 6 September 2023); Yun-Nan fire and rescue: https://weibo.com/u/2538642904 (accessed on 6 September 2023); AnHui fire and rescue: https://weibo.com/u/2524413584 (accessed on 6 September 2023); XinJiang fire and rescue: https://weibo.com/u/2486023042 (accessed on 6 September 2023); LuoYang fire and rescue: https://weibo.com/u/2451903763 (accessed on 6 September 2023); SanMing fire and rescue: https://weibo.com/u/2412731132 (accessed on 6 September 2023); China earthquake networks rapid report: https://weibo.com/u/1904228041 (accessed on 6 September 2023).

## Note

1 The Government Weibo Influence Report: The Weibo official releases an annual report on the influence and communication power of different government accounts, ranking them accordingly and the report ranks various government accounts.
